# Students at Risk: Self-Esteem, Optimism and Emotional Intelligence in Post-Pandemic Times?

**DOI:** 10.3390/ijerph191912499

**Published:** 2022-09-30

**Authors:** Lara Checa-Domene, Antonio Luque de la Rosa, Óscar Gavín-Chocano, Jorge Juan Torrado

**Affiliations:** 1 Department of Didactics and School Organization, Faculty of Education, University of Granada, Campus de Cartuja, s/n, 18071 Granada, Spain; 2 Department of Education, University of Almería, C/Carretera Sacramento, 04120 Almería, Spain; 3 Department of Pedagogy, University of Jaén, Campus las Lagunillas, s/n, 23071 Jaén, Spain

**Keywords:** university students, self-esteem, emotional intelligence, optimism, post-pandemic

## Abstract

Understanding the emotional profile of students during their training, as well as associated psychosocial factors such as optimism versus pessimism and self-esteem, is critical to improving student performance, especially in the post-pandemic period. In this study, 798 university students participated, belonging to the Degrees of Early Childhood and Primary Education, with a mean age of 24.52 years (±5.48). The following instruments were used: Wong Law Emotional Intelligence Scale (WLEIS-S), Life Orientation Test Revised (LOT-R) and the Rosenberg Self-Esteem Scale (RSES). The objective was to determine the predictive value of self-esteem on emotional intelligence and optimism vs. pessimism. A positive relationship between several dimensions of the instruments used (*p* < 0.01) were found. Moreover, the regression model predicted an association between emotional intelligence (use of emotions), pessimism and self-esteem. The practical consequences suggest the importance of the acquisition of emotional competences by university students is essential to obtain higher performances.

## 1. Introduction

It is undeniable that the situation generated by COVID-19 has, in general, provoked a major impact on the different components of our society. Particularly, in the academic sphere, most university students have manifested emotional disturbances in their daily lives, generating a great psychological impact that has led them, in the worst cases, to the edge of their own physical and psychological limits [1].

As a result of the effects caused by this pandemic, many and varied reflections have arisen on the importance of emotional intelligence in education, especially in university degrees related to teaching [2]. Several studies have corroborated the importance of identifying the emotional competences of trainee teachers in order to work on them, since teaching, according to Corbin et al. [3], is one of the professions recognised as the most stressful and, consequently, the one with the most emotional imbalances.

Similarly, the importance of promoting the development of emotional competencies in the university environment lies in the fact that it is a source of growth in the training process and in the achievement of the expectations of professional success of future teachers in a work context, that demands not only knowledge, but also to be bearers of emotional and social skills [4]. Therefore, the use of emotional regulation skills is essential and highly recommended not only to promote a positive and persistent emotional state in future teachers, but also to have the necessary strategies and tools to avoid possible psycho-emotional disorders in their professional field in order to be able to adjust in an efficient and effective way to the demands that the teaching profession urges.

From this perspective, and considering that future teachers will have a social responsibility when they begin to perform the profession to which they aspire, the concern arises to understand how this construct is related, in future teachers, to other variables such as self-esteem and optimism versus pessimism, since they directly and significantly influence not only the academic success and psychological well-being of the individual but also on his/her overall development and professional future career. In this way, the results obtained in this research will not only allow us to know the level of development of emotional competences and the correlation or discrepancy between variables, but will also contribute to the design of programs for the acquisition of the necessary emotional competences in the university context and in the future professional context, in order to face future work problems and their optimal solution.

### 1.1. Emotional Intelligence

In the study of emotional intelligence, many authors have examined this construct, adhering to different theoretical models, which has given rise to a wide variety of definitions [5].

Taking Salovey and Mayer’s model as a reference, due to its wide acceptance and empirical support in this field of research, EI is defined as the ability that allows individuals not only to perceive, assimilate and understand their own and others’ emotional states appropriately and accurately, but also to regulate and modify their mood or that of others [6]. From this perspective, EI is understood as an appropriate interaction between emotion and cognition that allows individuals not only to improve their cognitive processes and behaviour, but also to adapt optimally to their environment [7].

Consequently, emotionally intelligent people will not only be more able to perceive, understand and manage their own emotions, but will also be more capable of extrapolating their abilities to perceive, understand and manage the emotions of others, allowing them to know themselves, relate to others and find solutions to life’s problems in a more optimal way. The studies carried out in this line are based on the assumption that a student with high EI will present a higher level of response to emotional problems and enjoy greater emotional well-being [8], as well as having higher levels of self-esteem and better adaptation to stressful situations. In this order of ideas, it should also be noted that there is currently a strong belief, supported by the scientific literature, that women have higher levels of emotional intelligence than men in this context [4].

Likewise, authors such as Bisquerra, Extremera and Fernández Berrocal [9,10], state that they will also show less tendency to suppress thoughts, less depression and less tendency to anxiety or personal disorders.

### 1.2. Self-Esteem

Due to that self-esteem directly influences the development of the emotional skills of emotional intelligence, it has been selected to form part of the relevant pattern assumed in this research. According to Rosenberg [11], it is understood as the integral attitude that the person has towards him/herself in order to recognise and accept his/her own abilities, knowledge, feelings and bodily characteristics, whether positive or negative.

In the academic context, the level of self-esteem has a direct relationship with the level of emotional intelligence, as this has a positive or negative influence on the perception that the person has of himself, because it is the basis of his thoughts, feelings, beliefs and even his attitude. A person who scores high in self-esteem levels has a greater degree of confidence in his or her own value as a person, improving his or her self-concept and therefore, the self-efficacy to face different situations. In contrast to this, the lower the level of self-esteem, the more the subject devalues the value he/her has of themselves and their own abilities, developing a negative and distorted self-concept. 

Naranjo [12] states that a person with high self-esteem tends to act with confidence in their own judgement without excessive concern for the past and the future, being able to solve problems without being invaded or manipulated by the fears and personal and social insecurities they feels. In other words, recognising pleasant and unpleasant feelings and knowing how to manage them appropriately. On the other hand, in people with low self-esteem, a changing, unstable and vulnerable identity is observed. 

Furthermore, in the context of the present research, studies such as those by Lledó et al. [13] suggest that students with positive self-esteem have higher intrinsic motivation not only to cope with everyday problems but also to achieve higher academic achievement and, consequently, greater emotional stability, in contrast to those with negative self-esteem.

### 1.3. Optimism versus Pessimism

For its part, emotional intelligence contributes, in the same way, to the optimistic or pessimistic perception we have of everyday life events. The study of optimism began as a way of explaining the coping responses to negative events that happen to people in their lives externally to the subject, thus postulating the existence of two explanatory styles: optimistic and pessimistic [14]. 

From a theoretical perspective, both constructs have been approached from two different perspectives, the first one being Peterson and Seligman’s (1984) Explanatory Optimistic Style and the second one, Scheier and Caver’s (1987) Dispositional Optimism. For the present research, the second explanatory model is assumed since optimism is conceived as a dispositional personality trait. The model developed by these authors assumes that, when difficulties arise, favourable expectations increase people’s efforts to achieve goals, whereas unfavourable expectations reduce or nullify such efforts; thus, there is growing evidence that dispositional optimism and pessimism have opposite effects [15].

In this sense, several studies affirm that optimism can be explained as a person’s predisposition to attribute negative events to external causes to the subject, unstable over time and in specific life situations. Pessimism, on the other hand, can be understood as a predisposition to explain that negative events occur due to causes internal to the individual, which remain stable over time and can be extended to various areas of life. In this way, optimistic people maintain positive expectations when faced with adversity, the opposite of what happens to pessimistic people.

Thus, optimistic people use the necessary strategies to achieve their goals because they expect to achieve them, while pessimists believe that their results will always be negative. In addition, previous studies suggest a positive effect of optimism on behavioural coping and on the development of emotional experiences, showing that optimistic students have high levels of emotional intelligence and self-esteem. However, pessimistic undergraduates tend to experience higher levels of negativity, lower self-esteem and consequently poor management of emotional intelligence skills [16].

For all these reasons and based on the background defined in the theoretical framework, this study seeks to know in depth the levels of emotional intelligence, self-esteem and optimism of future teachers after the pandemic period. Therefore, the general objectives of this study are as follows: (a) to study the existence of statistically significant correlations between the dimensions of the EI assessment instruments (WLEIS-S), dispositional optimism (LOT-R) and self-esteem; (b) to establish the existence of statistically significant differences between the dimensions of the instruments considered and the socio-demographic variables (gender, university degree and age); (c) to analyse the predictive value of self-esteem with optimism, pessimism and the EI variables.

## 2. Materials and Methods

This quantitative study was conducted with a descriptive, comparative, correlational and cross-sectional design between the variables of EI, optimism and self-esteem in order to determine the correlations and differences between different dimensions and variables. Based on these criteria, longitudinal and reliability measures were established through Cronbach’s alpha and Omega coefficient, which is also known as Jöreskog’s *Rho*.

### 2.1. Participants

The sample is made up of 798 university students, belonging to the Early Childhood Education (*n* = 500) and Primary Education (*n* = 298) degrees of the Faculty of Educational Sciences of Almería, Granada and Jaén (Andalusia, Spain). For their selection, a non-probabilistic incidental sampling was used. The distribution of participants by gender is as follows: 652 were women (62.65%) and 146 men (37.35%), in line with the gender ratio in education degrees in Spain. The age range was between 18 and 58 years, with a mean age of 24.52 years (±5.48).

### 2.2. Instruments

*WLEIS-S.* The Spanish version of the *Wong Law Emotional Intelligence Scale* (WLEIS-S) [17] was used to measure EI. It is based on the Wong and Law EI scale (WLEIS) [18] and includes 16 items and 4 dimensions: intrapersonal perception (evaluation of own emotions), interpersonal perception (evaluation of the emotions of others), assimilation (use of emotions) and emotional regulation. A Likert-type scale of 7 points (1 to 7 points) was used, with a validity and reliability in Spanish contexts of α = 0.91.

*Life Orientation Test Revised.* The Spanish version of *the Life Orientation Test Revised* (LOT-R) [19] was used to assess optimism-pessimism. In a scale that measures the degree of optimism and pessimism, it is estimated that the higher the rating, the greater the optimism; on the contrary, for pessimism, a lower rating implies greater pessimism.

*Rosenberg Self-Esteem Scale* (RSES) [20]. The Spanish adaptation [21] was used to measure self-esteem, that is, the feelings of self-respect and self-acceptance through 10 items. Five of the items are written in positive and the remaining five in negative. It has been adapted and validated in the Spanish population showing satisfactory psychometric properties.

### 2.3. Procedure

The ethical guidelines promoted by national and international regulations for conducting research with people were followed, through the completion of informed consent and guarantee of confidentiality and anonymity of the data obtained. The instrument was administered individually through the Google^®^ platform (Google forms). The approximate response time for each student was 15 min. This research was approved by the Ethics Committee of Research on Human Beings of the University of Jaén (code OCT.20/1.TES).

### 2.4. Data Analysis

Descriptive statistics (means and standard deviations) were obtained, analysing a priori the validity, reliability (Cronbach’s alpha and Omega coefficient) and internal consistency of each instrument through confirmatory factor analysis (CFA), in order to verify the psychometric properties of the questionnaire and to obtain the factor loadings of each item. The normality analysis was performed by multivariate hypothesis testing (being the distribution of the multivariate normal set, each of the marginal variables will meet the criteria of univariate normality, but not vice versa), resulting in a non-normal distribution. The analyses were carried out using the IBM SPSS 25.0 software and the Jamovi software in its version 1.6 (Computer Software). In relation to the coefficients considered in this study, the Chi-square test (χ^2^), the degrees of freedom (gl), and the CFI, GFI, SRMR and RMSEA fit indices were used. In this sense, χ^2^ should be understood from the ratio in relation to the degrees of freedom (χ^2^/gl), where the values should be between 2 and 5. The comparative fit index (CFI) calculates the relative fit of the observed model, whose value should be greater than 0.90 indicating a good fit. Similarly, a goodness-of-fit index (GFI) above 0.90 indicates the proportion of variance and covariance of the model data. Similarly, the standardised root mean square residual (SRMR), standardised means of the residuals, i.e., the difference between the observed and model matrix, being less than 0.10 indicates a good model fit. The root mean square error of approximation per degree of freedom (RMSEA), as a measure of discrepancy, should have results below 0.08 [22,23]. In all cases, a 95% confidence level was used (significance *p* < 0.05).

## 3. Results

In the first instance, it was verified if the data assumed the assumption of normality by performing Mardia’s multivariate test to contrast the asymmetry and kurtosis of the observed variables, showing that the data did not follow a normal distribution. The assumptions of multicollinearity, homogeneity and homoscedasticity were also analysed in order to verify that the resulting distribution met the criteria of dependence between variables.

From the data obtained with each of the instruments (Table 1), a confirmatory factor analysis (CFA) was performed to verify the validity and internal structure of each item.

*Emotional Intelligence Questionnaire* (WLEIS-S): the factor loadings for the items of this scale presented an adequate fit [17], χ^2^/df = 1.725, with CFI = 0.989, SRMR = 0.038, RMSEA = 0.043. The reliability of this scale was Cronbach’s α = 0.886 and McDonald’s ω = 0.888.

*Dispositional Optimism Questionnaire* (LOT-R): the factor loadings for the items of this academic procrastination and self-regulation scale showed a moderate fit [19], χ^2^/df = 2.547, with CFI = 0.967, SRMR = 0.037, RMSEA = 0.074. The reliability of this scale was Cronbach’s α for optimism = 0.741 and McDonald’s ω = 0.750 and for pessimism = 0.632 and McDonald’s ω = 0.645.

*Rosenberg Self-Esteem Scale* (RSE): factor loadings for the items of this self-esteem scale showed an adequate fit [20], χ^2^/df = 3.102, with CFI = 0.921, SRMR = 0.061, RMSEA = 0.080. The reliability of this scale was Cronbach’s α = 0.859 and McDonald’s ω = 0.878.

### 3.1. Relationship between Resilience Variables (Self-Acceptance, Life Acceptance and Personal Competence) and Attitudes towards Diversity and Violence

Table 2 shows the scores of the correlation matrix, descriptive statistics (mean and standard deviation), reliability analysis (Cronbach’s alpha and Omega coefficient), presenting, in general, an adequate level of reliability in each of the variables that make up the evaluation instruments.

When analysing each of the variables, a statistically significant positive relationship between the EI variables was observed, being the highest value between appraisal of own emotions and emotional regulation (*r*_(798)_ = 0.669; *p* < 0.001). Additionally, between optimism and use of emotions (*r*_(798)_ = 0.565; *p* < 0.001). Similarly, there is a significant inverse relationship between the variable pessimism and the rest of the variables, being the one with the highest value with optimism (*r*_(798)_ = 0.400; *p* < 0.001). In the same way, there is also a relationship between self-esteem and the rest of the variables, being the highest value the emotional regulation (*r*_(798)_ = 0.384; *p* < 0.001). 

### 3.2. Differences According to Socio-Demographic Variables

To analyse the differences related to the socio-demographic variable of gender, the Mann-Whitney U test was used for two independent samples (Table 3). The results indicating that there were statistically significant differences in the variables appraisal of others’ emotions (*Z* = −2.705; *p* = 0.007), with higher scores for women than for men. There were also statistically positive differences between the variable emotional regulation (*Z* = −2.360; *p* = 0.018), with slightly higher scores for men than for women.

To calculate the effect size for this non-parametric test, the value of *r* [*r* = *Z*/√*n*] was obtained (Table 4). The effect size was small in all cases (*r* < 0.2), according to Cohen’s criteria.

To analyse the differences according to age, three intervals were established (18–29 years, 30–43 years and 44–58 years) using the H test of Kruskal-Wallis (Table 4).

In the dimensions appraisal of own emotions (χ^2^ = 16.039; *p* = 0.001), Use of emotions (χ^2^ = 6.161; *p* = 0.046) and emotional regulation (χ^2^ = 14.379; *p* = 0.001), statistically significant differences were found in relation to age, with higher values in older subjects. The effect size, epsilon squared (*ε*^2^), is small in all cases.

To analyse the differences according to university degree, two intervals were established, Early Childhood Education and Primary Education, respectively, using the U test of Mann-Whitney U (Table 5).

In the dimensions appraisal of own emotions (*Z* = −3.082; *p* = 0.002), appraisal of others’ emotions (*Z* = −2.305; *p* = 0.021), and optimism (*Z* = −3.601; *p* = 0.001), statistically significant differences were found, with the highest scores in the Early Childhood Education degree.

### 3.3. Linear Regression Study: Personal Competence

In order to explore and quantify the predictive capacity of each of the variables of study on self-esteem, a linear regression analysis was performed (successive steps), whose results are shown in Table 6, verifying the absence of multicollinearity problems (tolerance values < 0.20; *VIF* > 4.00), with values between 2.978 and 3.9894. The results of the Durbin-Watson test indicated that there was independence of errors, with a value of 1.936. Being between 1 and 3, we accept the assumption.

The dimension included in the regression model explains 20.0% of the variance, with the use of emotions variable being the best predictor of self-esteem (*R* = 0.447; *R*^2^ *Corrected* = 0.195 *F =* 27.814) and inversely pessimism, with the t-value being statistically significant in the variables tolerant beliefs, rejection of violence, intolerance and justification of violence towards minorities and as punishment.

## 4. Discussion

The main objective of this study was to determine the predictive value of self-esteem on emotional intelligence and optimism versus pessimism in a sample of education students from the Universities of Almería, Granada and Jaén, Spain. 

In the first instance, the reliability of the scores of each of the instruments was verified through the calculation of Cronbach’s alpha and subsequently the Omega coefficient, the latter as a more appropriate estimate when there is disparity in the factor loadings of each item (tau-equivalence), by working with the weighted sum of each variable and overcoming the limitations that could affect the proportion of variance [24].

In relation to the first objective, to analyse the existence of significant correlations between the factors of the emotional intelligence assessment instruments (WLEIS-S), optimism vs. pessimism (LOT-R) and self-esteem (RSES), the results indicated a statistically positive correlation between variables; being negative with pessimism as expected. Different studies corroborate these results, stating that optimistic people have greater well-being and are capable of facing challenges successfully [25]; on the contrary, pessimistic people tend to believe that adverse circumstances will continue over time, they will not find the necessary resources or strategies to change the situation and they will be more dissatisfied [26]. In the university context, a premonitory optimistic or pessimistic attitude towards desired goals in the near future can be considered as a good predictor of higher or lower academic performance, personal growth and self-esteem [27].

According to the second objective, to establish the existence of significant differences in the variables emotional intelligence, optimism vs. pessimism and self-esteem with the socio-demographic variable of gender, age and university degree, significant differences were found in the variable optimism, being the highest values for women, compared to men, and in older students. Regarding the differences related to the university degree in relation to this variable, higher values were found in the Early Childhood Education degree. Different investigations identify differences between men and women, the latter being those who better manage and pay more attention to their emotions [17]. However, the greater number of women could condition the results obtained. 

Despite the evidence reported, it is necessary to point out that these results are conditioned by the characteristics of the sample and the structure of the test, as there are more women than men in this sample. However, these results are consistent with other studies [28] that have shown that women tend to suffer more depressive states because they generally perceive problems and adverse situations more intensely, with pessimistic thinking habits prevailing.

Finally, to determine which variables predict greater self-esteem, a linear regression analysis was performed. In our case, the regression analysis found a relationship between the use of emotions and self-esteem and a negative relationship with pessimism. It should be noted that despite the importance of the data obtained, the predictive model does not provide sufficient evidence to ratify this relationship; however, it does allow us to corroborate the importance of socioemotional factors on greater or lesser self-esteem [29].

## 5. Conclusions

The results obtained during this study have allowed us to analyse the existence of correlations and significant differences between the different dimensions of the EI assessment instruments, dispositional optimism and self-esteem as predictors of the impact that these variables have on the professional development of future Early Childhood Education and Primary Education teachers. In this sense, the contributions presented are useful and relevant for this field of application since, firstly, they allow us to understand the real perception in relation to the different variables of emotional intelligence within the university context as well as what emotional skills are acquired by trainee teachers in order to achieve a future affective and effective teaching practice. Secondly, these findings show that self-esteem is a relevant indicator of pessimism or optimism, so it is essential to promote actions within the university context that allow emotionally intelligent working in education, since the practical implications of these results reinforce the implementation of educational actions related to the acquisition of tools and strategies that allow providing future teachers with the necessary resources to develop adequate emotional management. 

However, despite the evidence reported, it is necessary to point out some limitations that this study presents, which have to be taken into consideration for the continuity of similar studies and for new lines of research related to this subject. On the one hand, one of the main limitations of the study was the type of sample with which we studied. A sample size conditioned by a higher number of women in comparison to the number of men, which in the first instance, would make it difficult to extend the results to other contexts. Moreover, it is also directly influenced by the horizontal segregation ratio when students choose universities studies related to education, which are mostly chosen by women [30].

On the other hand, this study was carried out exclusively with university students, so the results cannot be fully generalised to other population segments. Likewise, we worked with students from three public universities belonging exclusively to the autonomous community of Andalusia. Therefore, it is suggested to extend this work to the general population, different cultural contexts or age groups, as well as to other universities in order to obtain a greater representativeness of the university population and a much more exhaustive knowledge. The identification of emotional competencies as determining component of the professional development of future teachers, will facilitate the study of emotional intelligence influence, as well as other motivational and attitudinal variables.

## Figures and Tables

**Table 1:** Factor loadings.

Latent Factor	Indicator	α	ω	Estimate	*SE*	*Z*	*p*	*β*	*AVE*	*CR*
Appraisal of own emotions	SEA1	0.876	0.878	0.881	0.0605	14.55	<0.001	0.687	0.571	0.879
	SEA2	0.883	0.885	0.446	0.0579	7.69	<0.001	0.387		
	SEA3	0.886	0.888	0.442	0.0634	6.98	<0.001	0.337		
	SEA4	0.879	0.881	0.795	0.0653	12.18	<0.001	0.577		
Appraisal of others’ emotions	OEA1	0.881	0.884	10.079	0.0586	18.40	<0.001	0.788	0.503	0.823
	OEA2	0.877	0.880	0.537	0.0577	9.30	<0.001	0.448		
	OEA3	0.874	0.877	0.834	0.0691	12.07	<0.001	0.565		
	OEA4	0.871	0.873	0.923	0.0609	15.16	<0.001	0.682		
Use of emotions	UOE1	0.891	0.894	10.009	0.0571	17.67	<0.001	0.776	0.418	0.722
	UOE2	0.875	0.878	0.138	0.0508	2.72	0.007	0.134		
	UOE3	0.880	0.882	0.908	0.0694	13.09	<0.001	0.603		
	UOE4	0.878	0.881	0.812	0.0720	11.28	<0.001	0.532		
Emotional Regulation	ROE1	0.881	0.883	0.873	0.0663	13.15	<0.001	0.610	0.526	0.816
	ROE2	0.876	0.879	0.490	0.0499	9.81	<0.001	0.477		
	ROE3	0.870	0.871	0.841	0.0670	12.55	<0.001	0.599		
	ROE4	0.876	0.878	10.029	0.0554	18.58	<0.001	0.805		
Optimism	OPT1	0.697	0.698	0.667	0.0550	12.14	<0.001	0.619	0.749	0.897
	OPT4	0.561	0.561	0.894	0.0568	15.74	<0.001	0.811		
	OPT10	0.699	0.699	0.698	0.0542	12.87	<0.001	0.679		
Pessimism	PESS3	0.633	0.638	0.531	0.0676	7.85	<0.001	0.455	0.461	0.798
	PESS7	0.459	0.461	0.678	0.0612	11.08	<0.001	0.629		
	PESS9	0.499	0.500	0.933	0.0731	12.77	<0.001	0.740		
Self-esteem	SE1	0.875	0.881	0.827	0.0419	19.74	<0.001	0.831	0.545	0.903
	SE2	0.870	0.876	0.883	0.0397	22.23	<0.001	0.893		
	SE3	0.871	0.877	0.806	0.0385	20.95	<0.001	0.855		
	SE4	0.871	0.878	0.699	0.0425	16.45	<0.001	0.734		
	SE5	0.872	0.879	0.688	0.0411	16.74	<0.001	0.742		
	SE6	0.871	0.881	0.660	0.0480	13.75	<0.001	0.644		
	SE7	0.872	0.881	0.653	0.0485	13.46	<0.001	0.634		
	SE8	0.889	0.897	0.303	0.0563	5.37	<0.001	0.279		
	SE9	0.889	0.897	0.303	0.0563	5.37	<0.001	0.279		
	SE10	0.884	0.892	0.548	0.0498	11.00	<0.001	0.528		

Note: AF5: self-concept questionnaire (academic, social, emotional, family and physical); academic procrastination scale (academic procrastination and academic self-regulation); *SE*: standardised error; *Z*: Z-value in the estimate; *p*: *p*-value of *Z* estimate; *β*: standardised estimate; *AVE*: average variance extracted; *CR*: critical ratio.

**Table 2:** Internal consistency, mean, standard deviation and Spearman’s correlation.

Variable	*M (SD)*	(1)	(2)	(3)	(4)	(5)	(6)	(7)
Appraisal of own emotions (1)	5.20 (±1.14)	-						
Appraisal of others’ emotions (2)	5.71 (±0.82)	0.461 ***	-					
Use of emotions (3)	5.16 (±1.11)	0.546 ***	0.339 ***	-				
Emotional Regulation (4)	4.70 (±1.13)	0.669 ***	0.309 ***	0.539 ***	-			
Optimism (5)	3.56 (±0.87)	0.431 ***	0.236 ***	0.565 ***	0.408 ***	-	-	
Pessimism (6)	2.80 (±0.88)	−0.297 ***	−0.102 *	−0.355 ***	−0.247 ***	-0.400 ***		
Self-esteem (7)	2.25 (±0.86)	0.220 ***	0.143 **	0.384 ***	0.205 ***	0.218 ***	−0.376 ***	-

*Note:* (1) Mean = *M*, Standard deviation = *SD*; (2) * *p* < 0.05, ** *p* < 0.01, *** *p* < 0.001.

**Table 3:** Rank differences according to gender (U of Mann-Whitney Test).

Variables	Women (*n* = 652) *M (SD)*	Men (*n* = 146) *M (SD)*	*Z*	*p*	Effect Size (*r*)
Appraisal of own emotions	5.16 (±1.16)	5.42 (±1.01)	−1.715	0.086	0.2300
Appraisal of others’ emotions	5.77 (±0.81)	5.48 (±0.84)	−2.705	0.007 **	0.3461
Use of emotions	5.14 (±1.13)	5.25 (±1.01)	−0.877	0.380	0.0944
Emotional Regulation	4.64 (±1.15)	4.98 (±1.02)	−2.360	0.018 *	0.2994
Optimism	3.53 (±0.90)	3.70 (±0.72)	−1.368	0.171	0.1990
Pessimism	2.84 (±0.89)	2.65 (±0.86)	−1.468	0.137	0.2160
Self-esteem	2.27 (±0.86)	2.15 (±0.90)	−0.995	0.320	0.1373

Note: (1) * = *p* < 0.05; ** = *p* < 0.01. (2) The statistical effect size is expressed as Cohen’s value.

**Table 4:** Rank differences according to age (H of Kruskal-Wallis Test).

Variable	18–29 Years *M (SD)*	30–43 Years *M (SD)*	44–58 Years *M (SD)*	χ^2^	*p*	*ε*²
Appraisal of own emotions	5.12 (±1.33)	5.69 (±0.99)	5.76 (±0.83)	16.039	0.001 **	0.04349
Appraisal of others’ emotions	5.69 (±0.83)	5.80 (±0.71)	5.84 (±0.48)	0.510	0.775	0.00486
Use of emotions	5.14 (±1.12)	5.48 (±0.94)	5.61 (±1.00)	6.161	046 *	0.03481
Emotional Regulation	4.63 (±1.11)	5.07 (±1.08)	5.44 (±0.83)	14.379	0.001 **	0.03924
Optimism	3.54 (±0.87)	3.69 (±0.85)	4.00 (±0.76)	4.003	0.135	0.01787
Pessimism	2.80 (±0.88)	2.81 (±0.92)	2.25 (±0.87)	2.546	0.280	0.01478
Self-esteem	2.24 (±0.86)	2.40 (±0.87)	2.46 (±0.88)	2.269	0.322	0.02069

Note: (1) * = *p* < 0.05; ** = *p* < 0.01. (2) Statistical effect size is expressed with the epsilon square value (*ε*^2^).

**Table 5:** Rank differences according to gender (U of Mann-Whitney Test).

Variables	Early Childhood (*n* = 500) *M (SD)*	Primary Educ. (*n* = 298) *M (SD)*	*Z*	*p*	Effect Size (*r*)
Appraisal of own emotions	5.33 (±1.15)	5.01 (±1.09)	−3.082	0.002 **	0.2300
Appraisal of others’ emotions	5.78 (±0.83)	5.60 (±0.79)	−2.305	0.021 *	0.3461
Use of emotions	5.22 (±1.12)	5.08 (±1.08)	−1.265	0.206	0.0944
Emotional Regulation	4.75 (±1.15)	4.63(±1.10)	−1.120	0.263	0.2994
Optimism	3.68 (±0.88)	3.37 (±0.83)	−3.601	0.001 **	0.1990
Pessimism	2.83 (±0.92)	2.78 (±0.83)	−0.417	0.677	0.2160
Self-esteem	2.23 (±0.88)	2.28 (±0.84)	−0.458	0.647	0.1373

Note: (1) * = *p* < 0.05; ** = *p* < 0.01. (2) Statistical effect size is expressed as Cohen’s value.

**Table 6:** Linear regression analysis, criteria variable: self-esteem.

Criteria Variable	*R*	*R* ^2^	*R*^2^ *Corrected*	*F*	Predicting Variables	*β*	*t*
Self-esteem	0.447	0.200	0.195	27.814			
					Use of emotions	0.283	5.818 **
					Pessimism	−0.256	−5.274 **

Note: (1) ** = *p* < 0.01.

## Data Availability

Data are available on justified request to the corresponding author.
